# CMR-based T1-mapping offers superior diagnostic value compared to longitudinal strain-based assessment of relative apical sparing in cardiac amyloidosis

**DOI:** 10.1038/s41598-021-94650-2

**Published:** 2021-07-30

**Authors:** Dennis Korthals, Grigorios Chatzantonis, Michael Bietenbeck, Claudia Meier, Philipp Stalling, Ali Yilmaz

**Affiliations:** Department of Cardiology I, University Hospital Münster, Albert-Schweitzer-Campus 1, Building A1, 48149 Münster, Germany

**Keywords:** Cardiology, Medical research

## Abstract

Cardiac amyloidosis (CA) is an infiltrative disease. In the present study, we compared the diagnostic accuracy of cardiovascular magnetic resonance (CMR)-based T1-mapping and subsequent extracellular volume fraction (ECV) measurement and longitudinal strain analysis in the same patients with (a) biopsy-proven cardiac amyloidosis (CA) and (b) hypertrophic cardiomyopathy (HCM). *N* = 30 patients with CA, *N* = 20 patients with HCM and *N* = 15 healthy control patients without relevant cardiac disease underwent dedicated CMR studies. The CMR protocol included standard sequences for cine-imaging, native and post-contrast T1-mapping and late-gadolinium-enhancement. ECV measurements were based on pre- and post-contrast T1-mapping images. Feature-tracking analysis was used to calculate 3D left ventricular longitudinal strain (LV-LS) in basal, mid and apical short-axis cine-images and to assess the presence of relative apical sparing. Receiver-operating-characteristic analysis revealed an area-under-the-curve regarding the differentiation of CA from HCM of 0.984 for native T1-mapping (*p* < 0.001), of 0.985 for ECV (*p* < 0.001) and only 0.740 for the “apical-to-(basal + midventricular)”-ratio of LV-LS (*p* = 0.012). A multivariable logistical regression analysis showed that ECV was the only statistically significant predictor of CA when compared to the parameter LV-LS or to the parameter “apical-to-(basal + midventricular)” LV-RLS-ratio. Native T1-mapping and ECV measurement are both superior to longitudinal strain measurement (with assessment of relative apical sparing) regarding the appropriate diagnosis of CA.

## Introduction

Systemic amyloidoses comprise more than 30 different disorders characterized by an infiltrative deposition of misfolded protein in various organs such as skin, eyes, lung, liver, kidneys, nervous system and heart. Depending on the variety and degree of organ involvement, the respective clinical manifestation may differ^1^. Amyloidoses are classified based on the misfolded protein precursor—with light-chain (AL) amyloidosis and transthyretin (ATTR) amyloidosis being the most commonly found forms in case of cardiac amyloidosis (CA)^2^. Moreover, it is well known that cardiac involvement is associated with high morbidity and poor prognosis^3,4^. Hence, early and appropriate diagnosis of cardiac involvement is highly important in order to start a targeted therapy on time.

The accumulation of amyloid deposits in the extracellular space of the myocardium results in both increased biventricular wall thickness and ventricular stiffness^5,6^ that are hallmarks of this restrictive cardiomyopathy, eventually leading to rapid progressive heart failure. Furthermore, deposition of amyloid fibrils in the atrial and ventricular wall may cause conduction abnormalities and contributes to the high prevalence of ventricular arrhythmias and atrial fibrillation, increasing the risk of sudden cardiac death (SCD) and thromboembolism^7^.

In contrast to CA, hypertrophic cardiomyopathy (HCM) is most often determined by mutations of those genes encoding sarcomere proteins of the contractile apparatus^8,9^. These mutations cause a disorganized arrangement of myocyte hypertrophy (called myocardial disarray) as well as expansion of extracellular matrix, composed of interstitial and replacement fibrosis^10^. Such structural changes in turn result in increased wall thickness and noncompliance of the left ventricle. Clinically, most patients with HCM are asymptomatic or show just mild symptoms, but a subset will progress to suffer from symptoms of heart failure, chest pain and arrythmias with an increased risk of SCD^11^.

Cardiovascular magnetic resonance (CMR) has been established as an important diagnostic tool for the work-up of left ventricular hypertrophy (LVH) of unknown origin. In this context, different myocardial patterns of late gadolinium enhancement (LGE) allow to differentiate CA from HCM: While the LGE pattern in CA is rather diffuse, starts mostly from the subendocardial layer of the basal segments and eventually spreads to all myocardial layers and segments^12,13^, HCM is characterized by a patchy and more focally accentuated LGE pattern predominantly occurring in the most hypertrophic septal segments of the left ventricular myocardium^14,15^.

More recently, T1-mapping has emerged as a new diagnostic technique, which offers tissue characterization by measurement of the intrinsic T1 relaxation time of the myocardium^16^. T1-mapping before infusion of a gadolinium-based contrast agent (native T1) and thereafter (post-contrast T1) allows to determine the extracellular volume fraction (ECV) of the myocardium^17^. Both native T1 and ECV are useful tools for the work-up of hypertrophic cardiac phenotypes of unknown origin. Since CA is characterized by an extensive, diffuse amyloid infiltration of the extracellular space^18^ while HCM mostly shows focally accentuated interstitial fibrosis in hypertrophied septal segments, the increase in global native T1 and ECV values is consistently lower in HCM as compared to CA^19^.

Another popular approach in the evaluation of infiltrative cardiomyopathies such as CA is based on tissue tracking techniques that aim to measure myocardial deformation kinetics and patterns (strain). Both echocardiography- and CMR-based strain techniques have been evaluated in different cardiac diseases. Particularly, speckle tracking echocardiography (STE) and CMR feature tracking (FT) are the most validated and clinically used methods for strain analysis^20^. In principle, in both diseases (CA and HCM) all strain parameters are somewhat reduced in advanced cardiac phenotypes. However, whereas the physiological gradient of baso-apically diminishing strain is mostly preserved in case of HCM, a distinctive and unique pattern is mostly observed in case of CA, i.e. an inversed pattern of gradual strain increase from the basal to apical segments of the left ventricle (LV), known as “cherry on top” or “apical sparing” phenomenon^21,22^.

With this study, we assessed the diagnostic value of T1-mapping-based approaches in comparison to strain-based techniques regarding the appropriate diagnosis and differentiation of CA and HCM.

## Methods

### Study population

This is a comparative, monocentric, prospective study. All patients underwent a routine CMR study for work-up of suspected non-ischemic cardiomyopathy. The first study group (CA group) comprised *N* = 30 patients with histologically proven cardiac amyloidosis (including both AL and ATTR subtypes). The second study group (HCM group) comprised *N* = 20 patients with “conventional” HCM showing preserved LV ejection fraction (LV-EF) ≥ 50%, LV wall thickness ≥ 15 mm (that could not be explained by abnormal loading conditions) and absence of LV outflow tract obstruction. The third group (control group) comprised *N* = 15 patients in whom structural or functional cardiac abnormalities were ruled out and who presented with a low pre-test probability of CAD. Exclusion criteria comprised: (1) relevant valvular disease (at least grade 2 in echocardiography and/or at least moderate in CMR), (2) prosthetic valve, (3) permanent atrial fibrillation and (4) obstructive coronary artery disease. The local ethics committee (Ethikkommission der Ärztekammer Westfalen-Lippe) approved the study protocol and every patient gave written informed consent before enrolment.

### CMR acquisition

**Figure 1:**
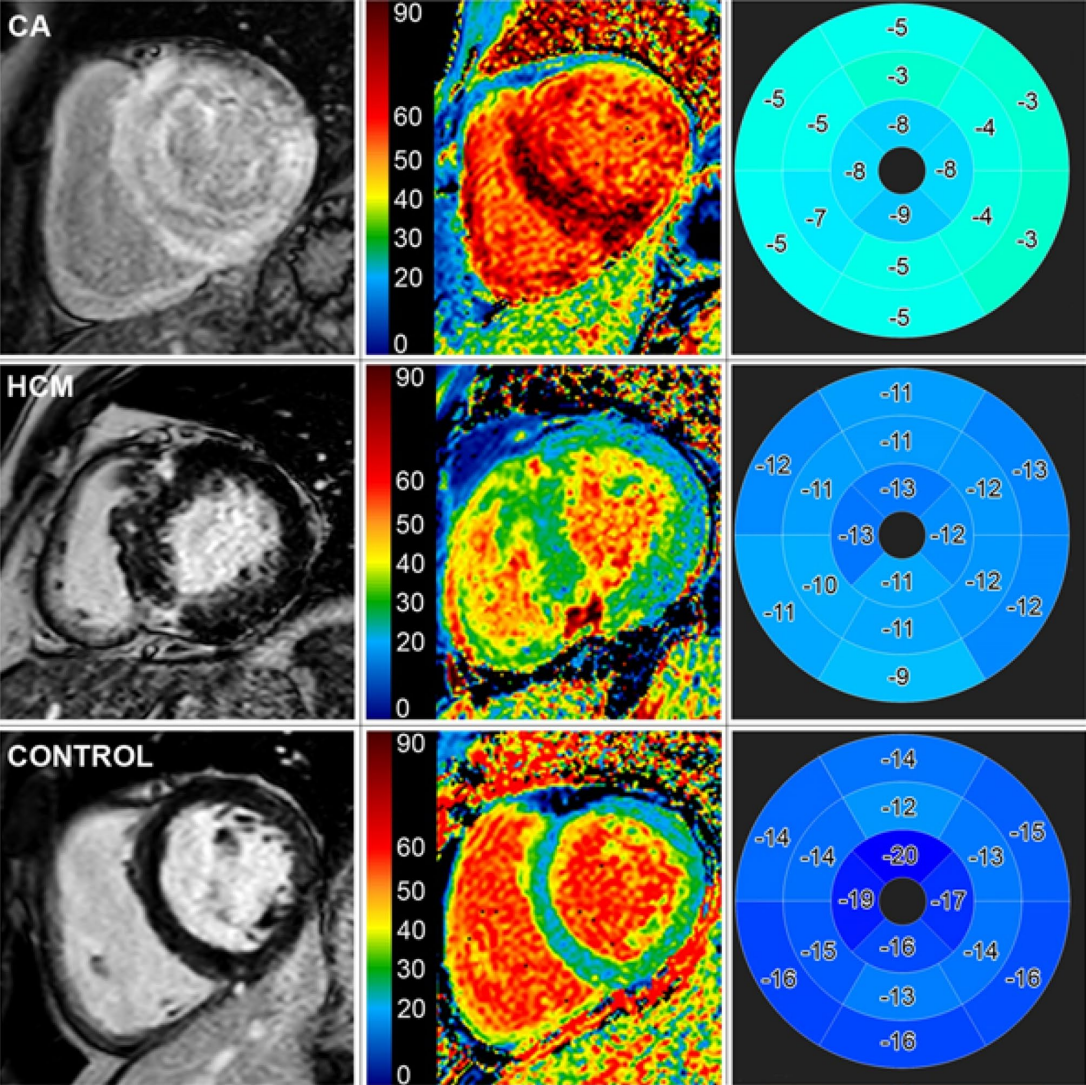
Late gadolinium enhancement (1st column), extracellular volume fraction maps (2nd column) and polar maps using a 16-segment model of peak longitudinal strain with values in percentage (3rd column) in short-axis views of a patient with cardiac amyloidosis (CA) (1st row), hypertrophic cardiomyopathy (HCM) (2nd row) and from the control group (3rd row).

All CMR studies were performed on a 1.5-T system (Ingenia, Philips Healthcare, Best, The Netherlands). CMR data acquisition was performed according to the standardized protocol suggested by the Society for Cardiovascular Magnetic Resonance (SCMR)^23^. Our CMR protocol comprised a cine steady-state free precession pulse sequence for ventricular function and a two-dimensional (2D) inversion recovery fast spoiled gradient-echo sequence 10 to 15 min after administration of gadolinium-based contrast (Gadobutrol 0.15 mmol/kg) for detection of myocardial pathology. Moreover, a modified Look-Locker inversion recovery (MOLLI) T1-mapping sequence was obtained in basal, mid and apical short-axes prior to contrast agent administration and ~ 20 min thereafter to determine native T1 and ECV values. Representative CMR acquisitions from all three groups of our cohort are illustrated in Fig. 1.

### CMR data analysis

Image analysis and interpretation was performed using commercially available software (cvi42—version 5.11.0, Circle Cardiovascular Imaging, Calgary, Alberta, Canada). Analysis of ventricular volumes and function as well as LV mass was made by contouring short-axis cine images. LGE images were visually assessed^24^ and the “Query Amyloid Late Enhancement” (QALE) score was reported as described in more detail elsewhere^25^.

### T1 and ECV measurements

T1-mapping and ECV were assessed and reported based on the consensus statement of SCMR^26^. Motion corrected native and post-contrast T1 maps were generated from the pre- and post-contrast T1 sequences. In each short-axis T1 map the endo- and epicardial contours were manually drawn. In order to further hamper contamination from blood pool and neighbouring tissues, respectively, a 10% safety margin was automatically set for both contours. Further, each short-axis map was automatically segmented (6 segments) using the RV insertion points as reference. Additionally, for ECV calculation, a region of interest was drawn in the blood pool (avoiding the papillary muscles) in all analysed T1 maps. Motion corrected and segmented ECV maps were generated from the native and post-contrast segmented T1 maps, using the patient’s haematocrit level.

### Feature tracking analysis

For the assessment of global LV deformation, three-dimensional (3D) LV global longitudinal strain (LV-GLS) derived from feature tracking (FT) was obtained using a validated algorithm integrated in the analysis software^27^. Landmarks for LV base (at the mitral valve ring) and apex were defined at end-diastole in all long-axis slices. Endocardial and epicardial borders were manually contoured in the end-diastolic frame in the three long-axis slices and in three short-axis slices, the most basal slice without through-plane distortion from the LV outflow tract, a mid-ventricular and an apical slice. Both the landmarks and the contours were automatically propagated throughout the cardiac cycle and manually corrected in case of inaccuracies. Subsequently, relative apical longitudinal strain (LS) was calculated based on the following equation: average apical LS/(average basal LS + mid LS), as defined by Phelan et al.^28^.

### Statistical analysis

Statistical analysis was performed with SPSS (version 25.0, IBM Corp., Armonk, NY). Continuous variables that showed a normal distribution are expressed as mean with ± standard deviation. Skewed variables are expressed as median ± interquartile range. Categorical variables are expressed as frequency with percentage. One-Way ANOVA with Bonferroni post hoc test was used for the comparison of normally distributed, homogenous data. When the assumption of homogeneity of variances was violated according to Levene’s test, Welch-ANOVA and Games-Howell multiple comparisons method were used instead. For the comparison of non-normal distributed data, we used the Kruskal–Wallis test. The Chi-square test with Bonferroni correction was used to compare categorical variables. Receiver operating characteristic curves (ROC) were analyzed to assess the diagnostic accuracy of different CMR parameters to differentiate CA from HCM patients. A univariable and subsequent multivariable logistic regression analysis was performed to identify the best predictor for the diagnosis of CA. A *p*-value < 0.05 was considered statistically significant.

### Ethics approval and consent to participate

The study protocol complies with the Declaration of Helsinki. Written informed consent was obtained from every patient.

## Results

### Study population

The study group characteristics are summarized in Table 1. Males and females showed a similar distribution in the CA and HCM group (77% in the CA group vs. 80% in the HCM group; *p* = 0.13). Median age differed significantly between the CA and both the HCM and control group due to the higher prevalence of CA in elderly patients. There were no other significant differences in major cardiovascular disease risk factors that could theoretically influence the results of this study.

**Table 1:** Patient characteristics.

	CA N = 30	HCM N = 20	Control N = 15	*p*-value
Male, n (%)	23 (77)	16 (80)	7 (47)	0.13
Age, years	69 (61–78)	49 (36–59)	30 (26–53)	**< 0.001**
Hypertension, n (%)	13 (43)	7 (35)	2 (13)	0.22
Diabetes, n (%)	2 (7)	3 (15)	0 (0)	0.43
High cholesterol, n (%)	12 (40)	4 (20)	1 (7)	**0.003**
Current smoker, n (%)	3 (10)	7 (35)	0 (0)	0.72

Bold values indicate significant *p*-value < 0.05.

### Conventional CMR findings

All anatomic, functional and structural CMR results are given in Table 2. Compared to HCM patients and controls, left ventricular ejection fraction (LV-EF) was slightly lower in CA patients (55 [± 9] % in CA vs. 65 [± 7] % in HCM, *p* < 0.001; and 61 [± 6] % in controls, *p* = 0.052)—but still preserved. The extent of LVH was similar in both CA and HCM patients (93 [85–105] g/m^2^ vs. 81 [62–99] g/m^2^, *p* = 0.25) with a more concentric pattern of LVH in CA compared to a more asymmetric, septally pronounced pattern in HCM patients. Furthermore, the distribution of LGE was characterized by a diffuse subendocardial to transmural pattern, predominantly affecting the basal to midventricular segments, and to a much greater extent in CA patients compared to the patchier, scattered pattern of LGE in HCM patients. Accordingly, the QALE score was significantly increased in CA compared to HCM (9 [6–12] vs. 2 [0–5], *p* = 0.001). No LGE was present in the control group.

**Table 2:** Conventional CMR results.

	CA N = 30	HCM N = 20	Control N = 15	*p*-value CA vs. HCM	*p*-value CA vs. control	*p*-value HCM vs. control
LV-EF, %	55 (± 9)	65 (± 7)	61 (± 6)	**< 0.001**	0.05	0.72
LV-EDV index, ml/m^2^	83 (± 16)	72 (± 14)	83 (± 12)	**0.040**	1.00	0.10
LV-ESV index, ml/m^2^	38 (± 11)	26 (± 8)	34 (± 9)	**< 0.001**	0.41	0.10
LV mass index, g/m^2^	93 (85–108)	81 (62–99)	55 (51–60)	0.25	**< 0.001**	**0.001**
Max. LV wall thickness, mm	18 (16–21)	17 (15–26)	9 (8–10)	1.0	**< 0.001**	**< 0.001**
RV-EF, %	54 (± 10)	65 (± 8)	57 (± 8)	**0.001**	1.0	**0.045**
RV-EDV index, ml/m^2^	79 (66–94)	64 (57–82)	82 (69–91)	**0.028**	1.0	**0.012**
RV-ESV index, ml/m^2^	36 (± 12)	25 (± 10)	37 (± 11)	**0.002**	1.0	**0.010**
LGE QALE score, n	9 (6–12)	2 (0–5)	0 (0)	**0.001**	**< 0.001**	**0.001**

Bold values indicate significant *p*-value < 0.05.

### T1-mapping and ECV findings compared to longitudinal strain

In patients with CA, global native T1 was significantly increased compared to both groups (1119 ms [1095–1174] in CA vs. (1014 ms [991–1051] in HCM, *p* < 0.001; and 983 ms [964–993] in controls, *p* < 0.001). Accordingly, global ECV was significantly higher in CA (50 [± 9] % in CA vs. 28 [± 10] in HCM, *p* < 0.001; and 28 [± 3] in controls, *p* < 0.001). Moreover, 3D-LV-GLS was reduced in both CA and HCM (− 6.7 [− 8.7 to − 4.9] % vs. − 10.5 [− 11.4 to − 8.1]), with a significant difference between these groups (*p* = 0.025). The most pronounced impairment of regional left ventricular longitudinal strain (LV-RLS) was measured in the basal segments of CA patients (− 5.7 [− 6.8 to − 3.6] % in CA vs. − 8.3 [− 12.1 to − 5.3] % in HCM, *p* = 0.036). The “apical-to-(basal + midventricular)”-ratio of LV-RLS (reflecting the degree of apical sparing) was 0.96 in CA vs. 0.72 in HCM (*p* = 0.004) and 0.065 in controls (*p* = 0.001), as depicted in Table 3.

**Table 3:** Novel CMR parameters.

	CA N = 30	HCM N = 20	Control N = 15	*p*-value CA vs. HCM	*p*-value CA vs. Control	*p*-value HCM vs. Control
**T1-mappping**						
Native T1-mapping global, ms	1119 (1095–1174)	1014 (991–1051)	983 (964–993)	**< 0.001**	**< 0.001**	0.30
Native T1-mapping basal, ms	1146 (± 68)	1024 (± 40)	985 (± 16)	**< 0.001**	**< 0.001**	**0.005**
Native T1-mapping mid, ms	1115 (1078–1171)	1021 (987–1057)	976 (965–991)	**< 0.001**	**< 0.001**	0.13
Native T1-mapping apical, ms	1141 (1074–1192)	994 (979–1045)	994 (962–1004)	**< 0.001**	**< 0.001**	1.0
**ECV**						
ECV global, %	50 (± 9)	28 (± 10)	28 (± 3)	**< 0.001**	**< 0.001**	1.0
ECV basal, %	53 (± 10)	28 (± 5)	27 (± 3)	**< 0.001**	**< 0.001**	0.64
ECV mid, %	49 (± 8)	28 (± 7)	27 (± 2)	**< 0.001**	**< 0.001**	1.0
ECV apical, %	50 (± 9)	29 (± 6)	29 (± 4)	**< 0.001**	**< 0.001**	0.99
**FT-strain parameters**						
Longitudinal systolic peak strain (3D) global, %	− 6.7 (− 8.7 to − 4.9)	− 10.5 (− 11.4 to − 8.1)	− 13.6 (− 14.9 to − 11.7)	**0.025**	**< 0.001**	**0.025**
Longitudinal systolic peak strain (3D) basal, %	− 5.7 (− 6.8 to − 3.6)	− 8.3 (− 12.1 to − 5.3)	− 12.9 (− 15.4 to − 10.9)	**0.036**	**< 0.001**	0.07
Longitudinal systolic peak strain (3D) mid, %	− 5.2 (− 6.9 to − 3.4)	− 8.9 (− 11 to − 6.8)	− 12.5 (− 14 to − 10)	**0.003**	**< 0.001**	0.07
Longitudinal systolic peak strain (3D) apical, %	− 10.4 (± 3.1)	− 12.9 (± 3.6)	− 16.4 (± 2.4)	**0.026**	**< 0.001**	**0.005**
Apical/(basal + mid) strain ratio (3D), n	0.96 (0.82–1.18)	0.72 (0.63–0.88)	0.65 (0.60–0.74)	**0.004**	**0.001**	0.74

Bold values indicate significant *p*-value < 0.05.

**Figure 2:**
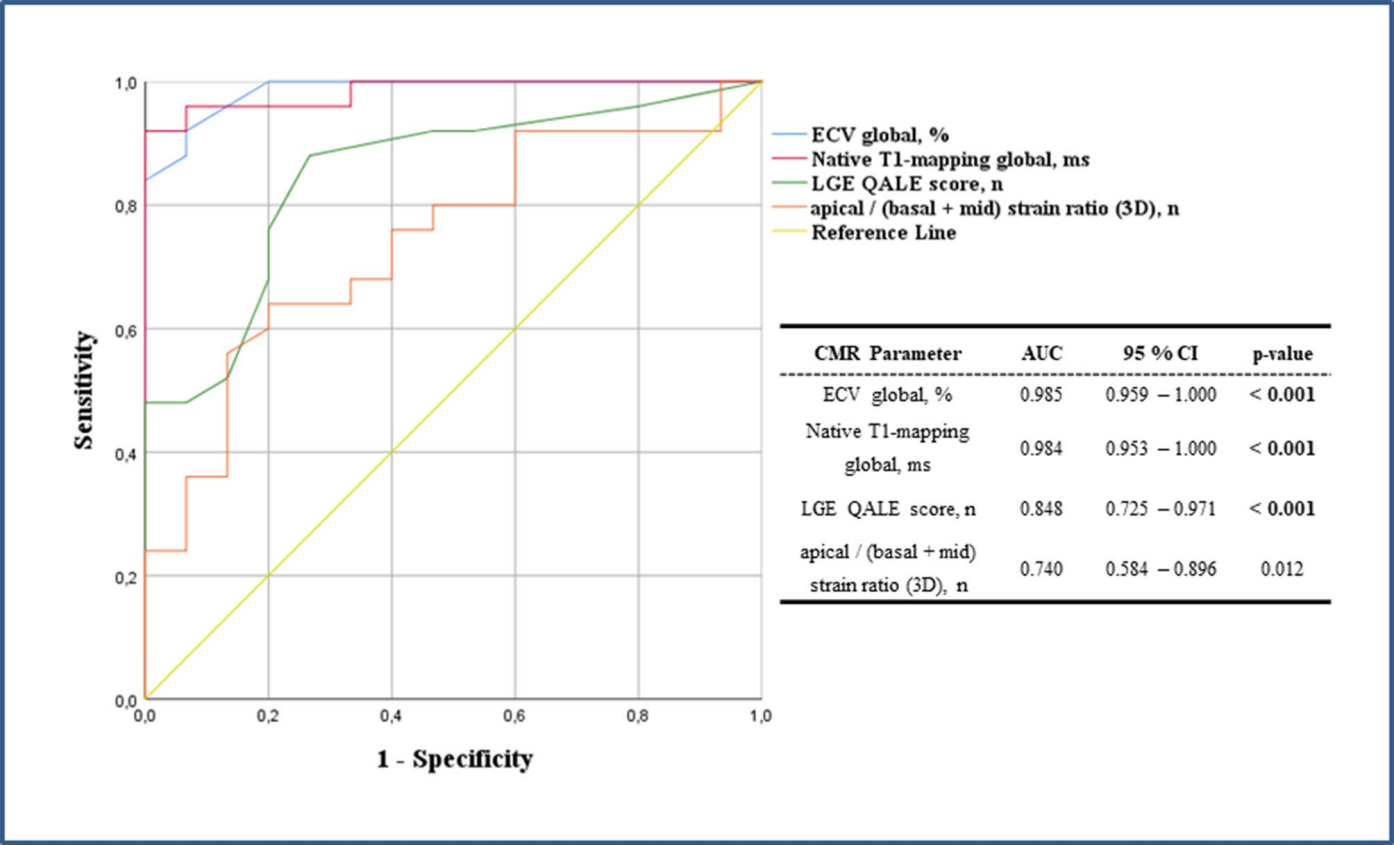
Receiver operating characteristic curve illustrating the diagnostic yield of the following parameters: i) extracellular volume fraction (ECV) global (light blue), (ii) T1-mapping global (red), (iii) Query Amyloid Late Enhancement (QALE) score (green), (iv) “apical-to-(basal + midventricular)”-ratio of left ventricular longitudinal strain (orange), in the distinction of cardiac amyloidosis (CA) from hypertrophic cardiomyopathy (HCM).

Receiver-operating-characteristic (ROC) analysis was performed for all novel CMR parameters, including native T1-mapping, ECV, “apical-to-(basal + midventricular)” LV-RLS-ratio plus the QALE score (Fig. 2). ROC analyses revealed that both native T1 and ECV provided the highest diagnostic accuracy regarding the differentiation of CA from HCM with an area-under-the-curve (AUC) of 0.985 for ECV (*p* < 0.001), of 0.984 for native T1-mapping (*p* < 0.001), of 0.848 for the QALE score (*p* < 0.001) and only 0.740 for the “apical-to-(basal + midventricular)” LV-RLS-ratio (*p* = 0.012). Finally, a multivariable logistical regression analysis showed that ECV was the only statistically significant predictor of CA when compared to the parameter 3D-LV-GLS or to the parameter “apical-to-(basal + midventricular)” LV-RLS-ratio (Table 4).

**Table 4:** Univariable and multivariable logistical regression analysis regarding the prediction of cardiac amyloidosis.

	Univariable analysis			Multivariable analysis		
	OR	95%-CI	*p*-value	OR	95%-CI	*p*-value
**A**						
ECV global (%)	1.335	1.108–1.609	**0.002**	1.362	1.090–1.700	**0.006**
Longitudinal systolic peak strain (3D) global	1.370	1.096–1.712	**0.006**	0.921	0.583–1.454	0.72
**B**						
ECV global	1.335	1.108–1.609	**0.002**	1.362	1.090–1.700	**0.006**
apical / (basal + mid) strain ratio (3D)	73.01	2.942–1812	**0.009**	1.584	0.010–252.1	0.86

Bold values indicate significant *p*-value < 0.05.

## Discussion

The present study focuses on a head-to-head comparison of the diagnostic performance of T1-mapping-based vs. strain-based CMR parameters in CA and HCM patients. Our study results clearly show that the novel CMR parameters native T1 and ECV show a higher diagnostic yield in the delineation of CA from HCM patients (as well as controls) compared to a strain-based approach assessing the presence of “apical sparing”.

Today, FT-CMR is a popular technique for strain analysis that tracks the displacement of individual patterns representing anatomical structures over time^29^. A major advantage of this technique compared to other strain approaches is its ease-of-use as the analysis can be performed very quickly on standard cine-CMR images after the examination. Balanced steady-state free precession (b-SSFP) sequences that are currently routinely used for standard cine imaging provide excellent blood-myocardium contrast as well as a high spatial and temporal resolution for motion tracking over the cardiac cycle. FT-CMR is most efficient in the detection of the endocardial border where the contrast between heart muscle and cavum is excellent. However, detection of the epicardial border can be challenging—in particular, when contrast between myocardium and surrounding tissue is poor. In some cases, tissue tracking may fail to find the transformation of every point within the myocardium because of the homogeneity of water content and properties of the tissue^30^. Moreover, it should be taken into account that a cine loop represents a reconstructed averaged cardiac cycle and thereby, in cases of high variation in heart frequency FT-CMR might give false measurements. In general, FT-CMR is a feasible and highly reproducible technique in various cardiac diseases. Noteworthy, assessment of LV-GLS by FT-CMR in case of LVH has shown good intra- und inter-observer variability with an intraclass correlation coefficient (ICC) of 0.82 (0.45–0.95) and 0.86 (0.54–0.96) respectively^31^. However, inter-study reproducibility seems to be poor (ICC: 0.44 [0–0.77])^32^.

Recently, a 3D FT-CMR approach was introduced and permits a complete 3D interrogation of myocardial strain. Theoretically, a 3D-based strain approach is more suitable for the assessment of global myocardial deformation since 2D techniques are prone to through-plane loss of features in the third dimension. Due to LV contraction (with concomitant rotation) during systole that results in movement of some segments outside the pre-defined imaging plane, overestimation of the observed degree of muscle shortening may occur^33^. It is for this reason that 3D FT-CMR measurements result in lower absolute values. Hence, in the present study, we used a 3D FT-CMR approach to obtain global as well as regional longitudinal strain and to calculate the degree of relative “apical sparing”.

Previous studies already showed that FT-CMR-based LV-GLS is significantly decreased in patients with CA compared to HCM^34^; a finding that is confirmed by our present results. Prior to the introduction of strain analysis using FT-CMR, speckle tracking echocardiography (STE) was already widely used for years. Based on STE studies in patients with CA, the concept of relative “apical sparing” (with regard to longitudinal strain) was introduced^28^: As longitudinal strain impairment in CA predominantly affects the basal segments of the LV—whereas apical segments are affected to a much lower degree, the concept of relative “apical sparing” was established and initially shown to differentiate CA from other causes of LVH with a sensitivity of 93% and a specificity of 82% by Phelan et al.^28^. In addition to such diagnostic data, a prognostic value of STE-based assessment of “apical sparing” was described in case of CA^35^ and this method was therefore even considered in current amyloidosis guidelines^36^. Since a good agreement was shown between STE-based and FT-CMR-based strain analysis, assessment of longitudinal strain was based on FT-CMR only in the present study^37^.

However, when Phelan et al. introduced the concept relative “apical sparing”, they used a cut-off value for relative “apical sparing” of 1.0 in order to differentiate CA from HCM with the aforementioned good sensitivity and specificity^28^. In contrast, in a more recent study, Pagourelias et al. obtained a poorer sensitivity of 63% and similar specificity of 85% for the diagnosis of CA using an optimal cut-off value of 0.87^38^. In our present CMR study, an optimal cut-off of 0.89 for the parameter “apical-to-(basal + midventricular)” LV-RLS-ratio resulted in a 64% sensitivity and 80% specificity for the diagnosis of CA—confirming the data of Pagourelias et al.^38^.

In contrast to echocardiography, CMR allows not only to assess myocardial strain but also structural changes using conventional LGE techniques and novel parametric mapping approaches such as native T1-mapping and T1-mapping-derived ECV. Native T1-mapping has the advantage of detecting subtle changes in the intrinsic properties of the myocardium that may be indicative of an early stage of cardiac disease. Moreover, in pathologies with diffuse or uniform pattern, T1-mapping has a superior diagnostic accuracy than LGE-imaging that requires relative signal intensity changes and reference regions of normal myocardium in order to depict tissue abnormalities^39^. Since extracellular amyloid deposition significantly increases native T1, accurate diagnosis of CA and differentiation from other causes of LVH is possible with T1-mapping: Recently, Baggiano et al. demonstrated the high diagnostic accuracy of both native T1 and ECV (AUC: 0.93 [0.92–0.96] and 0.99 [0.98–1.00], respectively) for the diagnosis of CA^40^. Moreover, the same group observed prognostic implications of T1-mapping-based analysis in CA patients—with ECV being independently predictive of death (hazard ratio [HR] 1.155 for each 3% increase; 95% CI 1.097 to 1.216; *p* < 0.001) in a multivariable Cox model that included (among others) N-terminal pro-B-type natriuretic peptide, LV-EF, LGE extent and LV mass^41^. Hence, both native T1 and ECV are highly sensitive non-invasive CMR parameters for the diagnosis of CA.

Surprisingly, an appropriate head-to-head comparison of the diagnostic performance of T1-mapping-based vs. FT-CMR-based parameters has not been performed in CA and HCM patients so far. To the best of our knowledge, this is the first study that provides a direct comparison of the diagnostic value between FT-CMR and T1-mapping in CA and HCM patients. In the present study, both native T1 and ECV substantially outperformed FT-CMR-based strain analysis with regard to the appropriate diagnosis of CA. Noteworthy, native T1 and ECV did not only demonstrate a substantially higher AUC based on ROC analyses compared to FT-CMR; in addition, ECV was an independent predictor of the presence of CA in multivariable regression analyses—in contrast to FT-CMR-based strain parameters. Hence, from a clinical point-of-view, strain-based assessment of relative “apical sparing” does not allow to safely rule out the presence of CA. Beyond strain measurement, novel CMR methods for myocardial tissue characterization such as T1-mapping and ECV measurement are required and should be routinely performed in patients with suspected CA and/or LVH of unknown origin. Moreover, a careful assessment of the diagnostic value of strain measurements is suggested in future recommendation and/or guideline papers addressing the diagnosis of CA.

### Limitations

Since CA is a rare disease, the size of our study group was limited^42^, however sufficient for the purposes of this study. In addition, CA consists of different subtypes dependent on the precursor protein. In the present study, no distinction was made between the different types of CA (AL and ATTR) since the underlying mechanism of cardiac involvement is similar^43^. Furthermore, the limitations of T1-mapping and ECV measurement^44^ as well as of FT-CMR are well documented^30^. Finally, we used a non-licensed dose of Gadovist (0.15 mmol/kg) for CMR imaging purposes and future studies need to show whether the licensed dose of 0.1 mmol/kg will be sufficient for tissue mapping purposes. However, based on previous data and according society recommendations^26^, our approach should be appropriate.

## Conclusion

Native T1-mapping and ECV measurement are both superior to longitudinal strain measurement (with assessment of relative apical sparing) regarding the appropriate diagnosis of CA. Hence, cardiac work-up of patients with suspected amyloidosis should be based on multi-parametric CMR including T1-mapping and ECV measurement—and not limited to echocardiography- and/or CMR-based longitudinal strain analysis.

## Supplementary Information

Supplementary Information 1.

Supplementary Information 2.

## Data Availability

The datasets used and/or analysed during the current study are available from the corresponding author on reasonable request.

## Abbreviations

AL: Immunoglobulin light chain amyloidosis
ATTR: Transthyretin amyloidosis
CA: Cardiac amyloidosis
CAD: Coronary artery disease
CMR: Cardiovascular magnetic resonance
DCM: Dilated cardiomyopathy
ECV: Extracellular volume
EMB: Endomyocardial biopsy
FT: Feature tracking
GLS: Global longitudinal strain
HCM: Hypertrophic cardiomyopathy
HF: Heart failure
LGE: Late-gadolinium-enhancement
LV: Left ventricle
LV-EDV: Left ventricular end-diastolic volume
LV-EF: Left ventricular ejection fraction
LS: Longitudinal strain
LVH: Left ventricular hypertrophy
SCMR: Society for cardiovascular magnetic resonance
QALE: Query amyloid late enhancement
