# Supplementary material for: CMR-based T1-mapping offers superior diagnostic value compared to longitudinal strain-based assessment of relative apical sparing in cardiac amyloidosis
Source: Sci Rep. 2021 Jul 30;11:15521. doi: 10.1038/s41598-021-94650-2 (PMC8324782; doi:10.1038/s41598-021-94650-2)
Supplement: Supplementary file 1 — Supplementary Information 1. [file 41598_2021_94650_MOESM1_ESM.pdf]

CMR-based T1-mapping offers superior diagnostic value compared to longitudinal strain-based assessment of relative apical sparing in cardiac amyloidosis

[1*]Dennis Korthals, [1*]Grigorios Chatzantonis, [1]Michael Bietenbeck, [1]Claudia Meier, [1]Philipp Stalling and [1]Ali Yilmaz

[1]Department of Cardiology I; University Hospital Münster; Münster, Germany

*: Both authors contributed equally to this work.

T1- / ECV-mapping

Like almost every other cvi42 circle user, we perform T1-/ECV-mapping according to the following steps. Further information can be found in the cvi42 manual or in the manual of the respective software provider.

1. Load native (T1n) and post-contrast series (T1e) in the respective module.
2. Draw endo- (green) and epicardial (red) contours as well as a blood (orange) contour and a marker for RV-Insertion (blue dot).

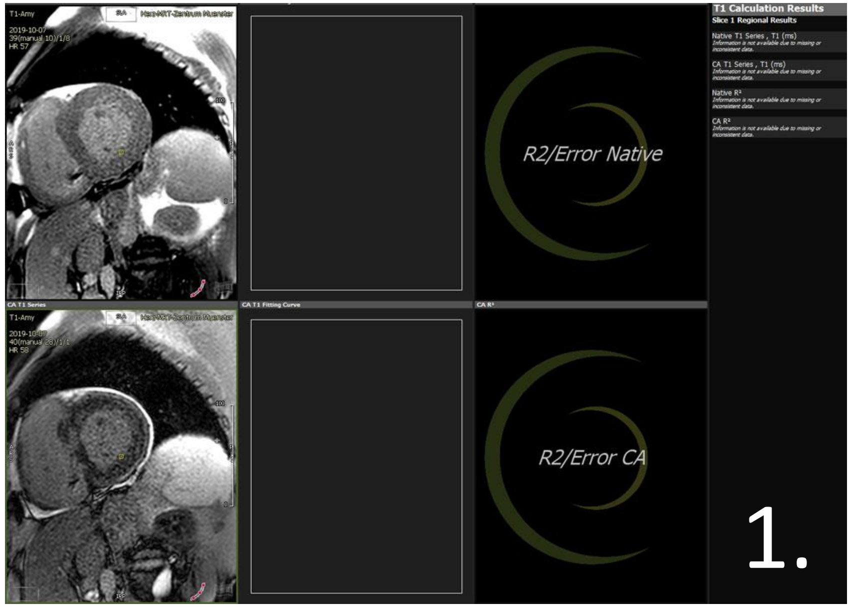

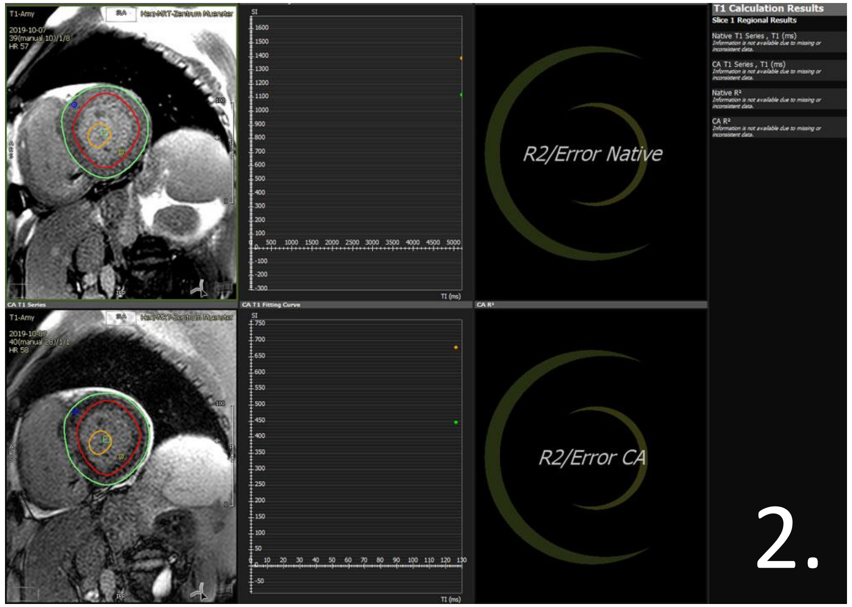

T1- / ECV-mapping

3. Forward the contours to all single images in T1n and T1e sequences.
4. Check for coarsely mismatched contours/myocardium and correct manually.

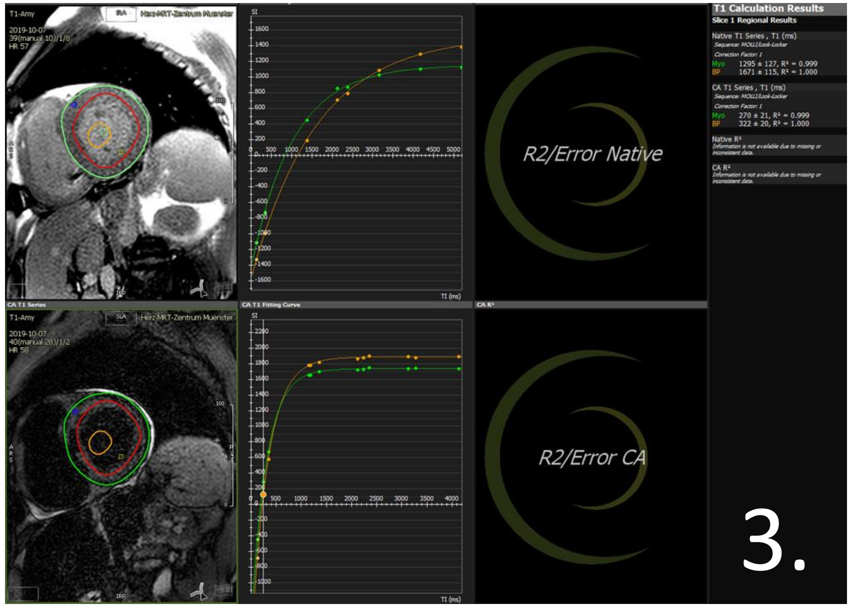

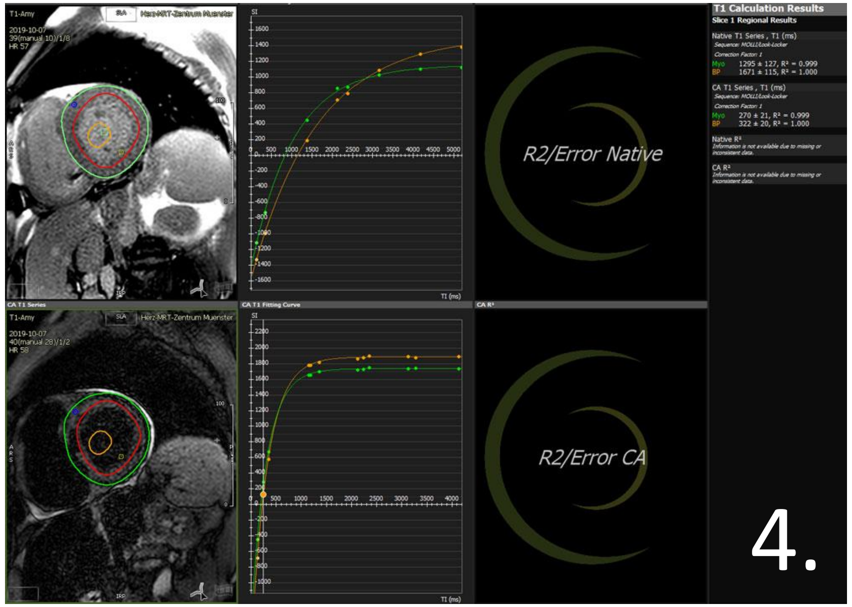

T1- / ECV-mapping

5. Register T1n- and T1e-images.
6. Generate T1n- and T1e-maps – copy contours.

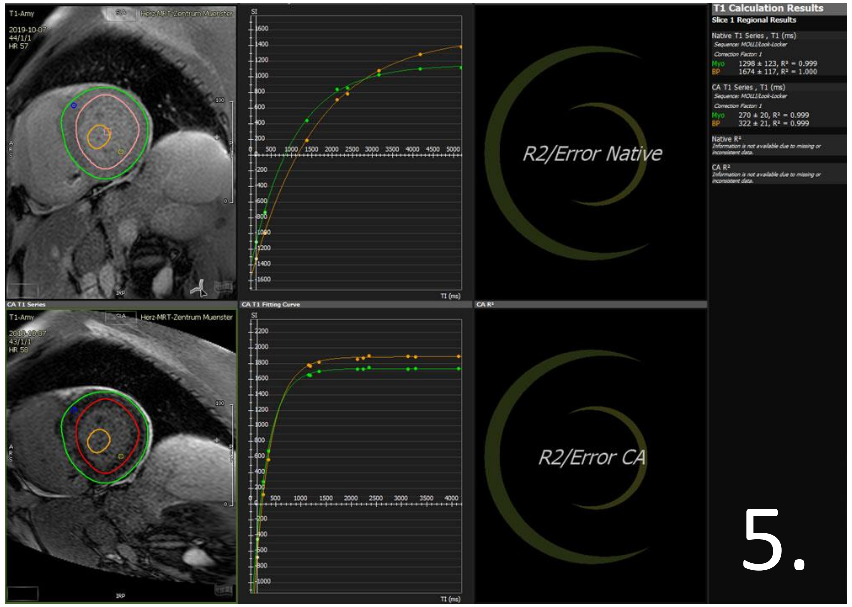

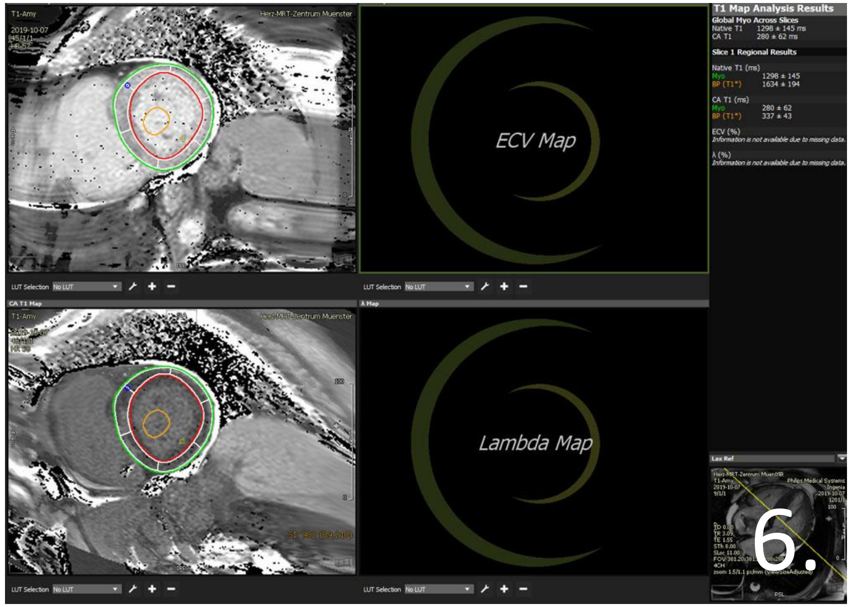

T1- / ECV-mapping

7. Enter recent hematocrit.
8. Calculate ECV-maps.

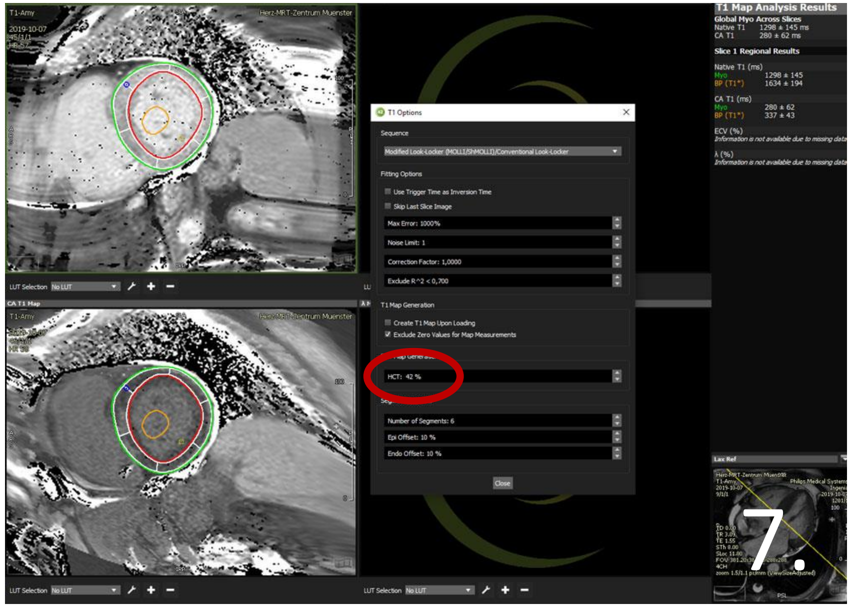

T1n-map

ECV map

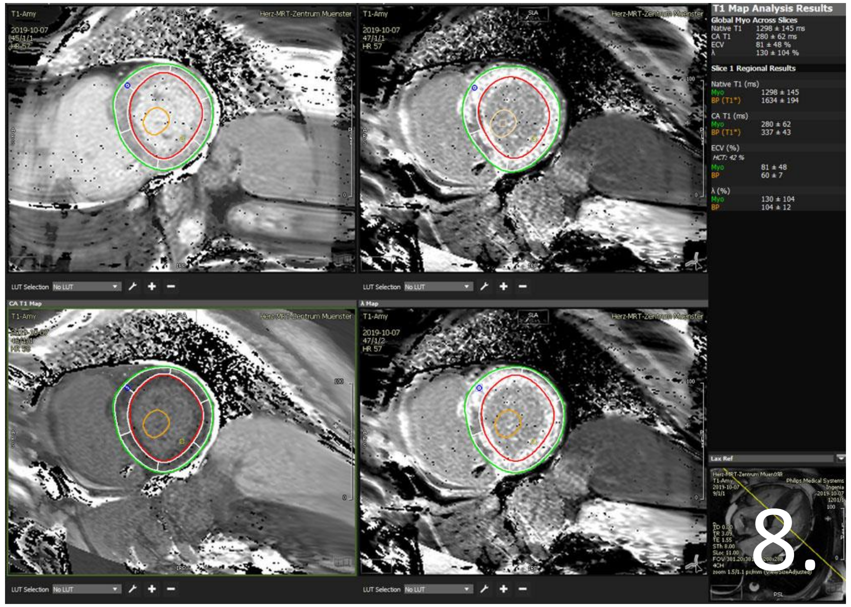

T1e-map

Global:
T1n: 1298 ms
T1e: 280 ms
ECV: 81 %
