# Supplementary material for: CMR-based T1-mapping offers superior diagnostic value compared to longitudinal strain-based assessment of relative apical sparing in cardiac amyloidosis
Source: Sci Rep. 2021 Jul 30;11:15521. doi: 10.1038/s41598-021-94650-2 (PMC8324782; doi:10.1038/s41598-021-94650-2)

CMR-based T1-mapping offers superior diagnostic value compared to longitudinal strain-based assessment of relative apical sparing in cardiac amyloidosis

[1*]Dennis Korthals, [1*]Grigorios Chatzantonis, [1]Michael Bietenbeck, [1]Claudia Meier, [1]Philipp Stalling and [1]Ali Yilmaz

[1]Department of Cardiology I; University Hospital Münster; Münster, Germany

*: Both authors contributed equally to this work.

(3D) cardiac magnetic resonance (CMR) feature tracking (FT) myocardial strain

1. In order to be sure that on the basal segments there is no underestimation in the strain analysis of the septal segments, we exclude the slices that contain the left ventricular outflow tract (LVOT).

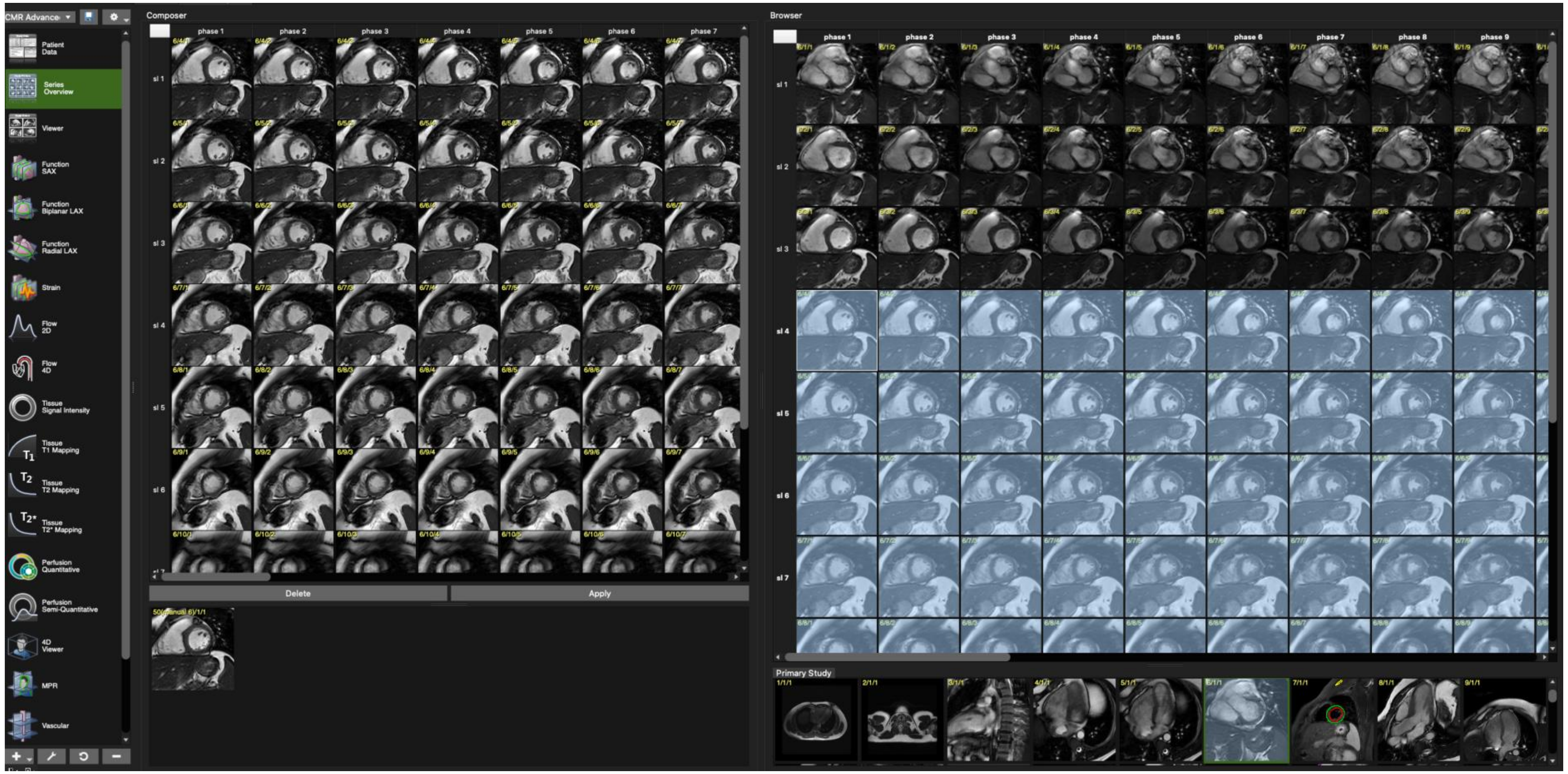

(3D) cardiac magnetic resonance (CMR) feature tracking (FT) myocardial strain

2. We then load the short axis (SA) stack and three long axis (LA) slices (4-, 3-, 2-chamber) of steady state free precession (SSFP) cine sequences in the respective module.
3. We draw endo- (red) and epicardial (green) contours in end-diastole.
4. For the contour delineation we use the same phase in the SA and LA series.
5. We set the anterior and inferior SA reference points at the insertions of the right ventricle.

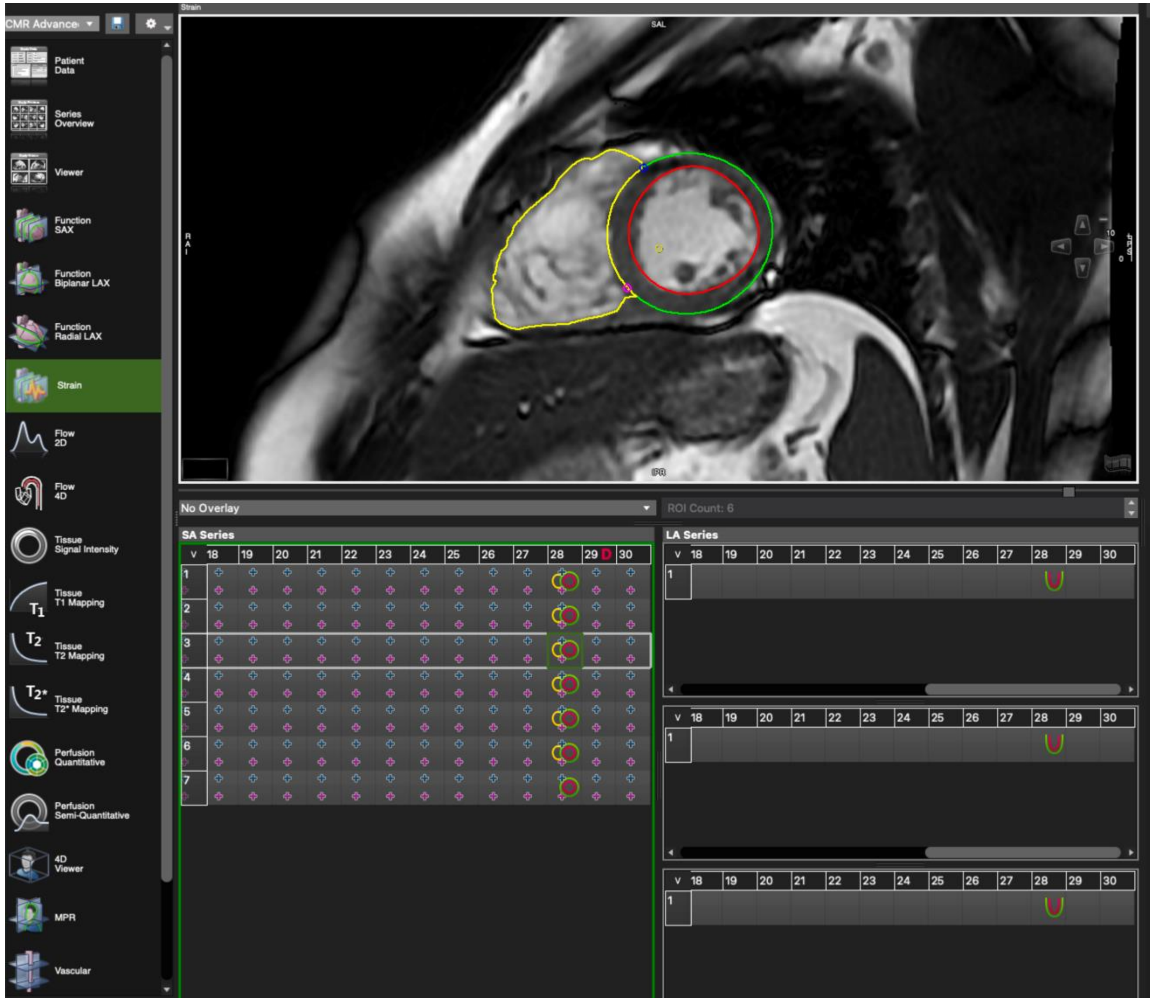

(3D) cardiac magnetic resonance (CMR) feature tracking (FT) myocardial strain

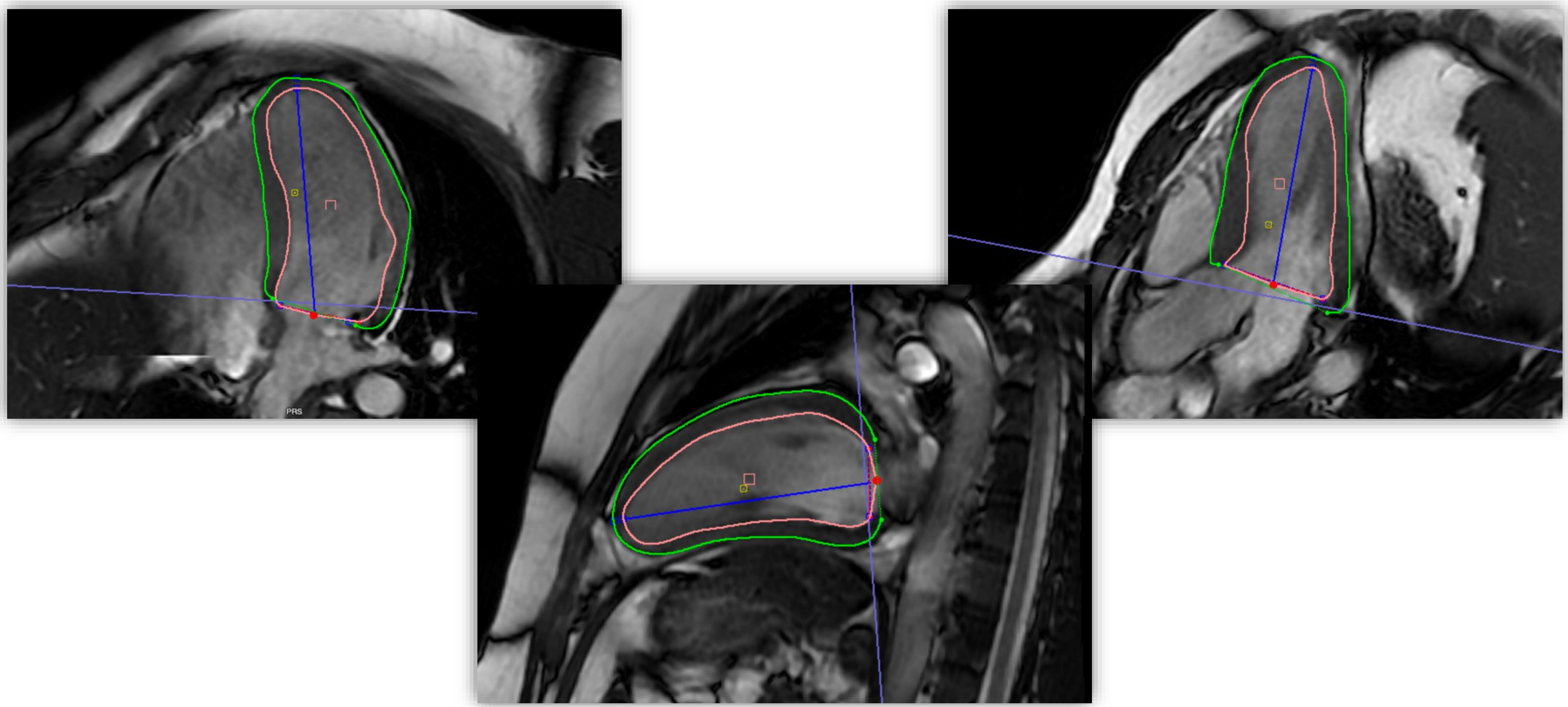

(3D) cardiac magnetic resonance (CMR) feature tracking (FT) myocardial strain

6. We select the reference phase and click the *Perform Automatic Strain Analysis button*.

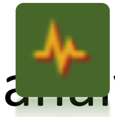

7. We perform the a[illegible]ysis of the *Longitudinal Strain* with *Basal-Mid-Apical Segmentation.*

8. We calculate the relative apical sparing of longitudinal strain accordingly to *Phelan et al. Heart. 2012;98(19):1442-1448. doi:10.1136/heartjnl-2012-302353*

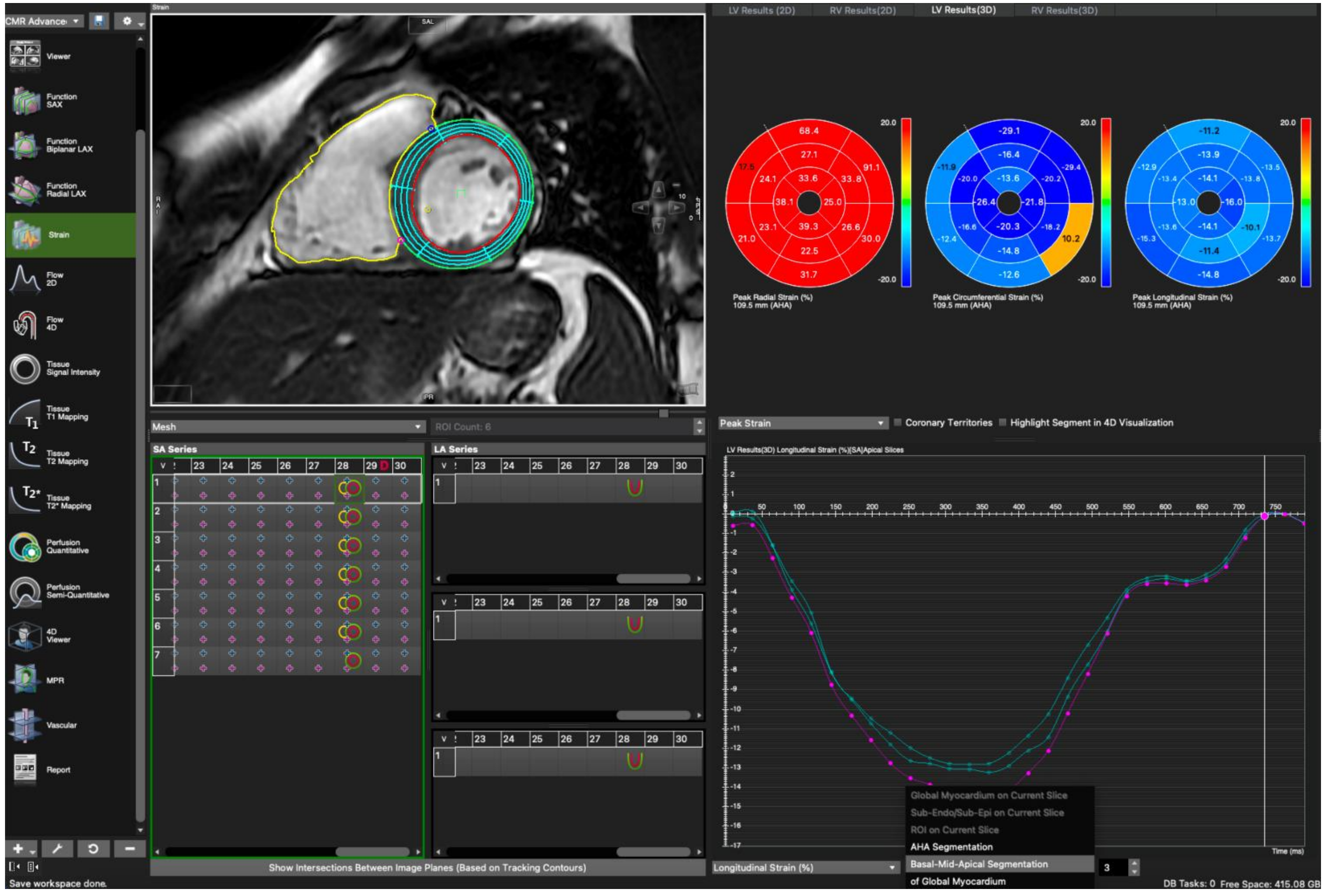

Supplement: Supplementary file 2 — Supplementary Information 2. [file 41598_2021_94650_MOESM2_ESM.pdf]
